# Identification of Diabetic Retinopathy Using Weighted Fusion Deep Learning Based on Dual-Channel Fundus Scans

**DOI:** 10.3390/diagnostics12020540

**Published:** 2022-02-19

**Authors:** Grace Ugochi Nneji, Jingye Cai, Jianhua Deng, Happy Nkanta Monday, Md Altab Hossin, Saifun Nahar

**Affiliations:** 1 School of Information and Software Engineering, University of Electronic Science and Technology of China, Chengdu 611731, China; ugochinneji@std.uestc.edu.cn (G.U.N.); jianhua.deng@uestc.edu.cn (J.D.); 2 School of Computer Science and Engineering, University of Electronic Science and Technology of China, Chengdu 611731, China; mh.nkanta@std.uestc.edu.cn; 3 School of Management and Economics, University of Electronic Science and Technology of China, Chengdu 611731, China; altabbd@uestc.edu.cn; 4 Department of Information System and Technology, University of Missouri St. Louis, St. Louis, MO 63121, USA; snnnm@umsl.edu

**Keywords:** CLAHE, CECED, deep learning, fundus scan, diabetic retinopathy, image identification

## Abstract

It is a well-known fact that diabetic retinopathy (DR) is one of the most common causes of visual impairment between the ages of 25 and 74 around the globe. Diabetes is caused by persistently high blood glucose levels, which leads to blood vessel aggravations and vision loss. Early diagnosis can minimise the risk of proliferated diabetic retinopathy, which is the advanced level of this disease, and having higher risk of severe impairment. Therefore, it becomes important to classify DR stages. To this effect, this paper presents a weighted fusion deep learning network (WFDLN) to automatically extract features and classify DR stages from fundus scans. The proposed framework aims to treat the issue of low quality and identify retinopathy symptoms in fundus images. Two channels of fundus images, namely, the contrast-limited adaptive histogram equalization (CLAHE) fundus images and the contrast-enhanced canny edge detection (CECED) fundus images are processed by WFDLN. Fundus-related features of CLAHE images are extracted by fine-tuned Inception V3, whereas the features of CECED fundus images are extracted using fine-tuned VGG-16. Both channels’ outputs are merged in a weighted approach, and softmax classification is used to determine the final recognition result. Experimental results show that the proposed network can identify the DR stages with high accuracy. The proposed method tested on the Messidor dataset reports an accuracy level of 98.5%, sensitivity of 98.9%, and specificity of 98.0%, whereas on the Kaggle dataset, the proposed model reports an accuracy level of 98.0%, sensitivity of 98.7%, and specificity of 97.8%. Compared with other models, our proposed network achieves comparable performance.

## 1. Introduction

Diabetes is caused by an accumulation of glucose in the bloodstream [1]. Diabetes puts a person at risk for various ailments, such as renal failure, loss of eyesight, teeth bleeding, nerve failure, lower limb seizure, stroke, heart failure, and so on [2]. Diabetic neuropathy is caused by the destruction of kidney nephrons, while diabetic retinopathy is caused by the injury in the brain neurons, which leads to retinal infection and can progressively impair eyesight at an early stage [3]. As a result, diabetic individuals must have comprehensive eye examinations during which the retina has to be examined by an ophthalmologist. Optical coherence tomography, fundus fluorescein angiography, slit lamp biomicroscopy, and fundus imaging are some of the methods used to identify the afflicted eye [4].

In accordance with the survey conducted by the World Health Organization (WHO), diabetes [5] is the seventh most deadly disease. Furthermore, with the supplementary statistics, there has been a high increment of diabetic patients which climbed up to 422 million. According to data, the number of diabetes-afflicted people over the age of 18 has increased from 4.7 percent to 8.5 percent, while some of the poorest persons are more likely to get diabetes. The maximum increase in glucose level has a significant impact on blood vessels, causing seeping of blood from the eyes and weakening of the human visual system [6]. Humans, on the other hand, are born with the power to cure the sickness. When the brain recognizes blood leaking, it stimulates the surrounding tissues to deal with the situation. As a result, it causes the sporadic formation of new blood vessels, but the resulting cells are anemic [7].

Retinal fundus image analysis is a helpful medical processing operation. Ophthalmologists can employ retinal blood vessel segmentation to help them diagnose a variety of eye problems [8]. As a result, diseases including diabetic retinopathy, hypertension, atherosclerosis, and macular degeneration can alter the morphology of the arteries, thereby producing alterations in their diameter, tortuosity, and branching angle. Manually segmenting retinal vascular diseases is a time-consuming and skillful operation.

The severity of the illness can be determined by the abnormal size of any afflicted body component. There are a few forecasting models that are regarded as important concepts, such as exudate, venous beading, microaneurysms, and hemorrhaging. Microaneurysms are blood clots that are 100–120 µm in diameter and have a round form [9]. Hemorrhage [10] is produced by a large quantity of blood leaking from a damaged blood vessel. Neovascularization is the term for the unequal expansion of blood vessels. Non-proliferative DR (NPDR) and proliferative DR (PDR) are the two types of DR. As a result, the DR sample indicates various levels, as illustrated in Table 1.

Early prediction of DR can play a significant role in preventing vision loss. Further, the structural change as a result of the vascular system may provide physical signs for the disease; hence, medical specialists advise patients to receive annual retinal screening tests utilizing dilated eye exams [11]. Interestingly, these retina scans might be used to detect diabetes, although this would necessitate ophthalmologists’ general judgment, which could take time.

Deep Learning techniques have demonstrated superior performance in the identification of DR, with a high level of accuracy which distinguishes them from other models. Undoubtedly, DL can uncover hidden elements in images that medical specialists would never see. Due to its capability in feature extraction and training in discriminating between multi-classes, the convolutional neural network (CNN) is the most commonly used DL approach in the medical system [12]. On several medical datasets, the transfer learning (TL) approach has also made it easier to retrain deep neural networks quickly and reliably [13,14].

Several machine vision applications are so complex that they cannot be solved with just one algorithm, which has prompted the design of models that incorporate two or more of the methodologies investigated. The weighted fusion strategy involves more than one model to tackle this challenge, since models are selected based on the problem’s specifications and feature extraction. By combining the features derived from a single model in a weighted manner, this procedure was developed to help single models mitigate their defects and enhance their strengths. This technique decreases prediction variance while reducing generalization error. As a result, the purpose of this research is to evaluate the effectiveness of weight-fusing neural network models for identification of DR which aids in the reduction of vision loss caused by DR and reduces the stress and time-consumption of ophthalmologists.

The remaining sections of this article is outlined in the following manner: Section 2 discusses the related work of DR algorithms; Section 3 explains the method behind our suggested approach; Section 4 presents the experimental findings and model evaluation. Section 5 presents the discussion of our study; and lastly, a conclusion is written in Section 6.

## 2. Literature Review

In hospitals, medical practitioners carry out a comprehensive dilated eye exam, where drops are put in a patient’s eyes, allowing the examiner to see inside the eyes more clearly and examine for abnormalities. Fluorescein angiography is another diagnosis method involving the injection of yellow dye called fluorescein into a vein in a patient’s arm. This dye passes through the blood vessels and into the body [2]. As the dye flows through the retina’s blood vessels, a unique camera captures pictures of it. This determines whether any blood vessels are clogged or leaking fluid, along with the amount of edema in the macula. It also reveals whether or not any aberrant blood vessels are forming. OCT angiography is a modern technology that examines blood arteries without the use of dye. Optical coherence tomography (OCT) is a type of diagnosis method that uses light to create images (OCT). The images produced by this test provide cross-sectional images of the retina, which reveal the thickness of the retina. This will assist in identifying how much fluid has leaked into the retinal tissue, if any at all. OCT tests may then be used to track how well the therapy is functioning [8].

Additionally, some research articles have presented DR diagnosis based on precise lesions or clinical indications, for example, Glucose, LDL-Cholesterol, and HbAIs [11]. An analysis by the Ref. [11] identified risk factors and ranked HbAIs, LDL-cholesterol, and glucose as the most influential risk factors. Utilising these factors, machine learning models were developed to identify the diabetic patient from non-diabetic patients. Aslan et al. [15] proposed a preprocessing and filtering conversion strategy to achieve fundus scan segmentation by making the blood vessel more noticeable when extracting the features. The Top-Hat transform, Adaptive threshold, and Gabor filter were the specific preprocessing procedures adopted. According to the authors, their approach had a high accuracy rate of 94.59%.

Further, some researchers have provided reports using the deep learning framework. Gulshan et al. [16] suggested the utilization of Inception V3 to detect RDR trained on 128,000 images of fundus and obtained 99.1% and 97.5%, respectively. Shankar et al. [5] introduced the HPTI-v4 diagnostic model for classifying DR, which achieved a very high accuracy level of 99.49%, specificity level of 99.68%, and sensitivity level of 98.83%. A strategy of the weighted fusion of pre-processed images for the classification of healthy and RDR was suggested by the authors in the Ref. [17] by utilizing the mixture of a residual network and decision tree algorithm. The authors reported 93% sensitivity, 94% AUC, and 87% specificity.

Shanthi et al. [18] reported an average accuracy level of 96.25% after making structural modifications to the AlexNet framework utilizing the Messidor dataset. DCNN was suggested by the authors of the Ref. [19] for the segmentation and detection of DR lesions. New biomarkers were revealed when heatmaps were applied to the DR images. The authors reported a 95% ROC value on the Kaggle dataset. With the utilization of the Messidor dataset, a study in the Ref. [20] suggested Inception-ResNet-V2 with the Moth optimization approach to extract features for classifying fundus images. The authors recorded 99.12% accuracy and 97.91% sensitivity.

A scheme that combines decision tree and bootstrap for the formulation of a double-channel process for segmenting fundus images was proposed in the Ref. [21]. A combined scheme of visual captioning and accelerated efficient properties which is based on CNN was developed by authors of the Ref. [22] to extract delicate local information. With the utilization of the Messidor dataset, the authors of the Ref. [23] incorporated an attention algorithm into a pre-trained model to find patch locations, commonly for DR detection. A re-scaling scheme called SI2DRNet-v1 was suggested by the authors in the Ref. [24], where the size of the kernel is scaled down. In the Ref. [25], the authors created a technique for locating blood veins along with a pretreatment for bound component analysis. The dimensionality was then reduced using linear separation analysis. In their technique, SVM was utilized for classification.

Additionally, the LeNet model was suggested by the authors of the Ref. [26] as a technique for EX detection. They disbanded the EX zones and transferred their training to the LeNet model. Before the training, they replicated the data. The Kaggle dataset was used to create the project. The authors of the Ref. [27] dealt with overfitting and skewed datasets in the context of DR detection. The CNN model, which has 13 layers, was trained using data amplification using the Kaggle dataset. Another paper used an ensemble method of CNN models [28] to identify all phases of DR using balanced and unbalanced classes. To begin with, they divided the Kaggle dataset into three sections and generated three sub-datasets. In the first model, they used DenseNet-121 to train three datasets independently and then combined the results. In the second model, they used ResNet50, DenseNet-121, and Inception-V3 to train three datasets independently and then combined their findings. The models were then compared to one another. It is worth mentioning that DL models have achieved significant results in medical imaging applications in recent times [29,30,31,32].

Though various DL models have been established for the classification of DR, enough emphasis has not been placed on the low resolution and quality which may influence the performance of DR classification. To this view, a weighted fusion deep learning network (WFDLN) is proposed for DR identification. Since the deep learning network has good performance in identifying images, two kinds of images are analyzed—one is CLAHE images, and the other is CECED images. We considered CECED and CLAHE because CECED provides and represents vital shape features in image classification and the CLAHE method limits histogram amplification and enhances the inverse of an intensity image. A pre-trained VGG-16 was built for the extraction of features from the CECED images, whereas Inception v3 was used for the extraction of features from CLAHE fundus images. Output from the dual channel fundus scans were then fused in a weighted pattern, and a Softmax classifier was used for the prediction of results.

Our paper focuses on the problem of low-quality DR images. The novelties of our proposed model is in threefold. First, the dual channels of fundus images which are CLAHE and CECED images are utilized for DR identification because of their interconnected properties. Secondly, the fine-tuning techniques are utilised for better extraction of features. Lastly, the output weights of each channel are merged for a robust prediction result. Two public datasets belonging to Kaggle and Messidor are used to evaluate the performance of our research.

## 3. Materials and Methods

The acquisition of data, image pre-processing, and networks for feature extraction and classification are the three phases of the proposed strategy. The procedure of the proposed approach in this paper is discussed in the subsequent subsections.

### 3.1. Datasets

**Figure 1:**
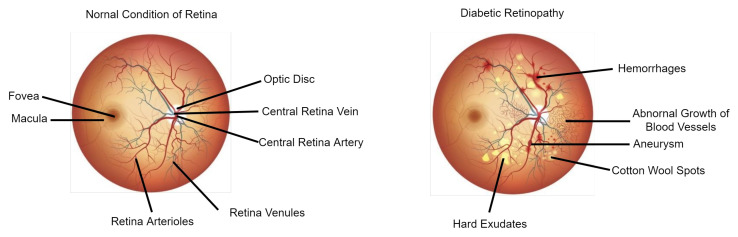
Illustration of the entire retina image. The fundus image on the left depicts the normal retina condition, and the fundus image on the right depicts a retina with diabetic retinopathy symptoms.

Comparing the performance of our proposed scheme, we applied two open-source datasets separately—the first is the Messidor dataset, and the second is the Kaggle dataset. Figure 1 shows both the healthy retina image and the unhealthy retina image showing diabetic retinopathy symptoms.

#### 3.1.1. Messidor Dataset

The Messidor dataset [33], as presented in Table 1, was collected from three ophthalmologic stations utilizing a digital video recording camera mounted on a Topcon TRC NW6, which is specifically a non-mydriatic retinograph with the specification of a 45-degree field of view to collect color pictures of 1200 fundus scans. The capturing resolutions of the pictures were 1440×960, 2240×1488, or 2304×1536 pixels using 8 bits per color plane. The dataset was classified into four phases—healthy ones were labeled as normal, images with microaneurysms were labeled as Stage 1, images with both microaneurysms and hemorrhages were labelled as Stage 2, and finally, images with significant microaneurysms and hemorrhages were labelled as Stage 3. More so. data augmentation was carried to reproduce a total of 2000 images for the Messidor dataset.

#### 3.1.2. Kaggle Dataset

The Kaggle dataset, as shown in Table 2, is also analyzed in this study. The dataset was acquired from the website of EyePACS for the Kaggle diabetic retinopathy competition [34], which contains 35,126 fundus images taken under various imaging circumstances. An expert categorized these fundus images on a scale of 0 to 4 depending on the intensity of DR. The five types of DR along with their proportions are given in Table 2. Out of the total number of datasets, we only selected 2000 images for our model implementation.

### 3.2. Image Pre-Processing

**Figure 2:**
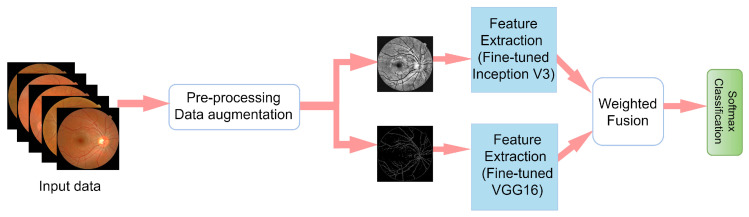
Pipeline of our proposed diabetic retinopathy identification based on a weighted fusion deep learning network.

The pipeline for our proposed approach is presented in Figure 2. First, the images were resized to 224×224 for the VGG-16 network channel and 299×299 for the Inception V3 network channel. Then, CLAHE and CECED were utilized as a pre-processing technique to create two different kinds of image sets.

#### 3.2.1. CLAHE Images

**Figure 3:**
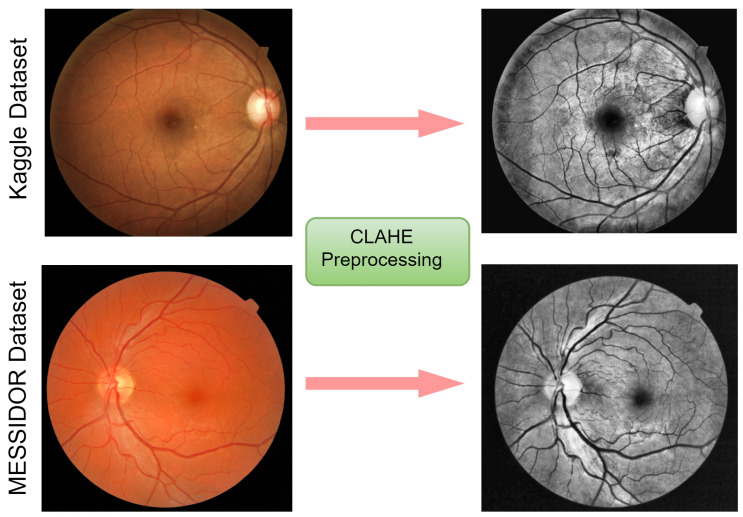
Visual representation of the fundus images from the Kaggle and Messidor datasets. On the left are images before pre-processing, and on the right are images after CLAHE pre-processing.

In this study, CLAHE was employed to enhance the contrast and characteristics of the image by making anomalies more visible. Among the histogram equalization-based family, CLAHE has a more realistic look in appearance and is useful in the reduction of noise distortion; therefore, we examined and utilized it in our dataset, as presented in Figure 3. A full explanation is given below to demonstrate its effectiveness:

- The first phase of the CLAHE scheme is the generation of image transformation by using the histogram bin value.
- Following that, using a clip border, the contrast is confined to a binary value from 0 to 1. A clip boundary is applied before the process of image segmentation.
- A precise bin score is created to prevent the background mapping areas to grayscale. To obtain good mapping, a histogram clip boundary is utilized.
- Lastly, the completed CLAHE image is generated by estimating the regions of the image, then extracting, mapping, and interpolating all pixel images to achieve optimal output.

#### 3.2.2. CECED Images

Applying the technique in the Ref. [35], the contrast enhancement and Canny edge detection technology were combined in the CEED-Canny approach. The steps are given below:

- Collect the value of the original pixel, along with the local minimum and maximum;
- Enhance the morphological contrast of the image;
- Apply Gaussian smoothing to reduce noise;
- Determine the image’s intensity gradient;
- Use a non-maximum suppression method; and
- Apply the hysteresis thresholding technique.

**Figure 4:**
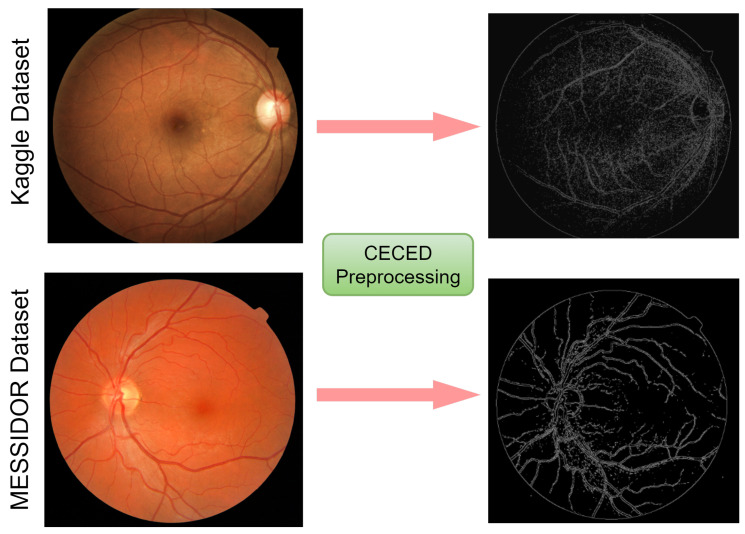
Visual representation of the fundus images from the Kaggle and Messidor datasets. On the left are images before pre-processing, and on the right are images after CECED pre-processing.

Edges are made up of important and relevant detailed information and characteristics, as seen in Figure 4. The quantity of data that have to be processed may be decreased, and the information deemed less important can be filtered out by using an edge detector on an image. We hypothesized in this study that a retina with DR will have more unusual edges than healthy retina. These characteristics may aid in the diagnosis of DR.

### 3.3. Feature Extraction

Two CNN architectures, VGG-16 and Inception V3, were used in this article. As a fixed feature extractor, the CNNs were pre-trained. A new model was created using the retrieved characteristics as an input. VGG-16 and Inception V3 were simply utilized as feature extractors, and their layers were either trained and/or frozen. To avoid overfitting, dropout layers were added.

### 3.4. Extraction of Features from CLAHE Images

**Figure 5:**
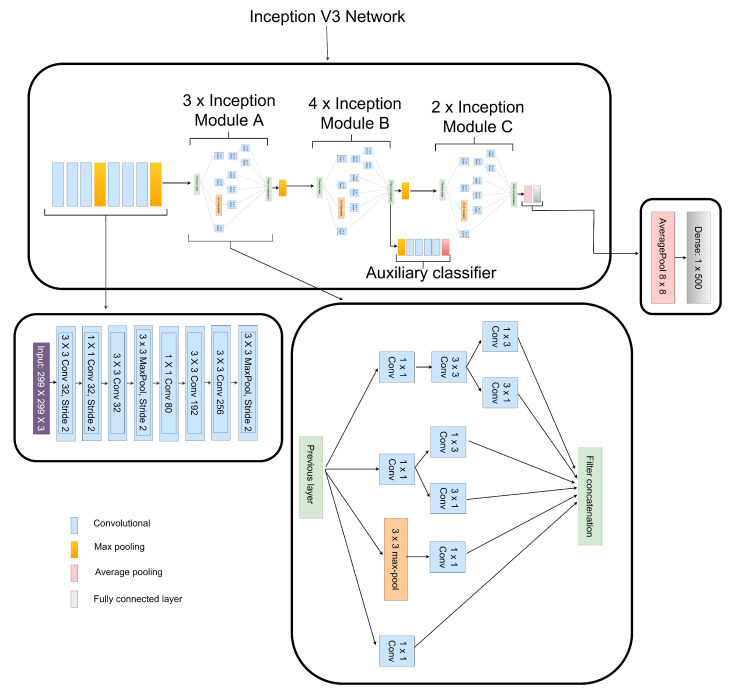
Structure of the fine-tuned Inception V3 network used to extract DR and non-DR related features from CLAHE fundus images.

Numerous network connection methods, like batch normalization, utilizing MLP convolutional layers to substitute linear convolutional layers, and factorizing convolutions with bigger kernel size, contribute to Inception V3’s excellent performance [36]. These methods substantially decrease the amount of network parameters along with computational cost, allowing the network to be constructed much deeply and with greater non-linear expressive capacity than traditional CNN models. We made a few modifications to the Inception V3 model by factorizing the 7×7 convolutional layer to 3×3, 1×1, and 3×3 instead of the original 3×3, 3×3 and 3×3 convolutional layers as presented in Figure 5. We also reduced the Inception module B to 4× instead of the original 5×, as seen in Figure 5. Our modifications reduced computational cost and achieved reduction in feature dimensionality during the low-level feature extraction and overall network depth. Average pooling of 8×8 was used instead of those which were conventionally fully connected to flatten the feature vector, according to the authors in the Ref. [36]. We preserved only one dense layer having its dimension set as 1×500, as seen in Figure 5.

### 3.5. Extraction of Features from CECED DR Images

**Figure 6:**
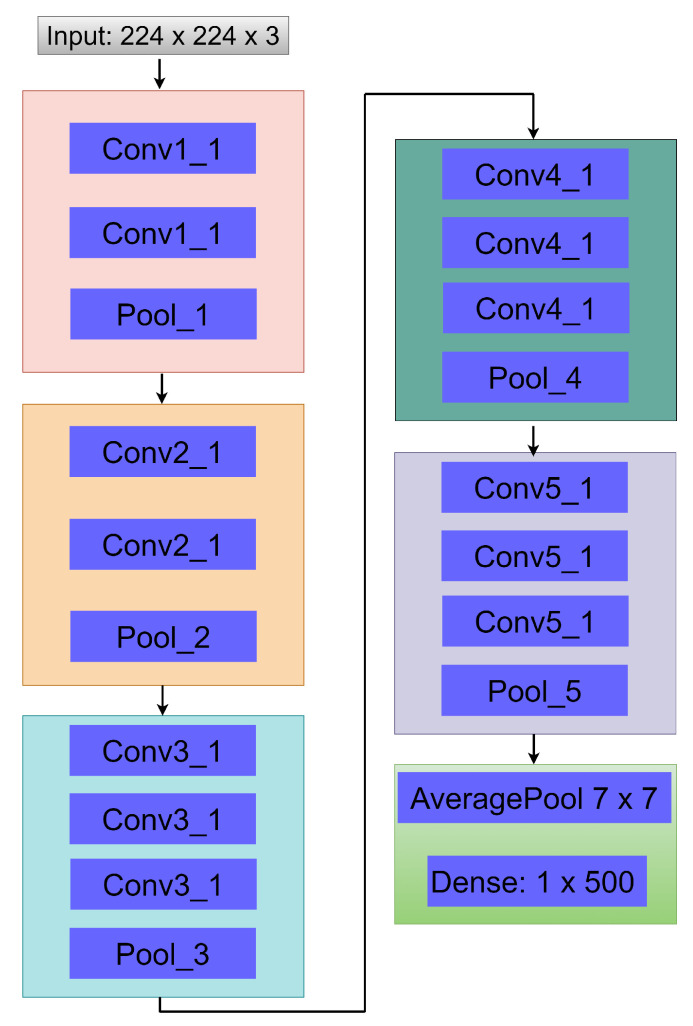
Structure of the fine-tuned VGG-16 network used to extract DR and non-DR related features from CECED fundus images.

In this extraction phase, we used the VGG-16 network due to its satisfactory performance in image classification, which converges quite fast [37]. A few modifications were made to simplify our network by removing two dense layers, so only one dense layer was left, having a dimension of 1×500 as seen in Figure 6. As a strategy to enhance the accuracy of our proposed approach, we utilized average pooling of 7×7 to achieve flattening instead of the conventional fully connected layer, as presented in Figure 6.

### 3.6. Weighted Fusion of the Different Output Channels

**Figure 7:**
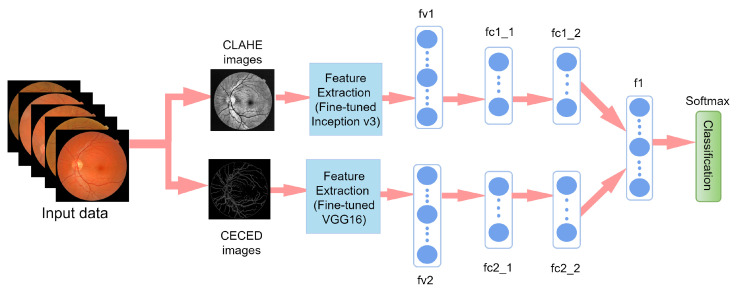
Our proposed weighted fusion deep learning network (WFDLN).

Figure 7 presents the proposed weighted fusion model. The feature vector, fv1, of the CLAHE fundus images was extracted using fine-tuned Inception V3 strategy. Feature vector fv2 was extracted from CECED fundus images using fine-tuned VGG-16. For the purposes of dimensionality reduction, each of the feature vectors was connected to one dense layer after flattening with average pooling. As presented in Figure 7, the fc1_1 and fc2_1 are the average pooling layers for fv1 and fv2, while fc1_2 and fc2_2 are the dense layers for fv1 and fv2. The network captures the distances between various retina characteristics and reveals them via fc1_2 and fc2_2. Furthermore, to create a fused vector, fc1_2 and fc2_2 were fused in a weighted manner into f1. Based on the fused feature vector, Softmax was utilized to classify fundus retina images into DR stages.

## 4. Experimental Results

Our proposed model is implemented on Keras framework with Python programming language using NVIDIA GTX 1080 GPU. A data split ratio of 70%, 20%, 10% for training, validation and test respectively while using Adam optimizer with batch size of 32, and learning rate of 0.0001. The metrics adopted as the evaluation criterion to examine the diagnostic performance of our proposed WFDLN were accuracy (ACC), sensitivity (SEN), precision (PRE), and specificity (SPE). The numerical expressions for each metric are presented in Equations (1)–(5) [38].

(1) $$Accuracy=\frac{TP+TN}{TP+TN+FP+FN}$$

(2) $$Sensitivity=\frac{TP}{TP+FN}$$

(3) $$Specificity=\frac{TN}{TN+FP}$$

(4) $$Precision=\frac{TP}{TP+FP}$$

(5) $$F1-score=2\times \frac{Precision\times Recall}{Precision+Recall}$$

TN denotes true-negative, TP stands for true-positive, FP depicts false-positive, and FN denotes false-negative.

### Evaluation of the Weighted Fusion

**Figure 8:**
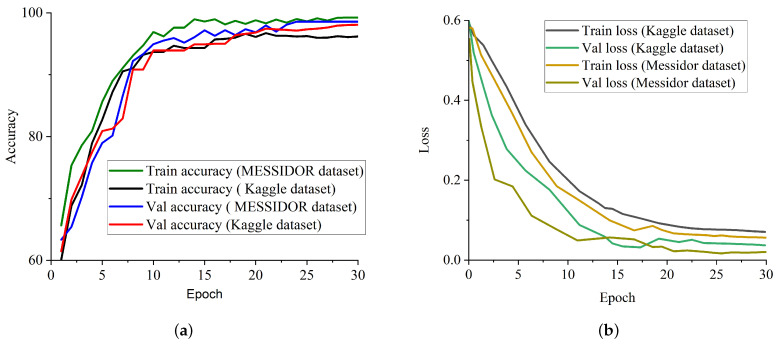
Training and validation performance report of our proposed model on both Messidor and Kaggle datasets. (**a**) Accuracy curve for our proposed model on both Kaggle and Messidor datasets. (**b**) Loss curve for our proposed model on both Kaggle and Messidor datasets.

We conducted some studies to evaluate the influence of our proposed weighted fusion method to the identification performance in terms of accuracy on two benchmark datasets. The first study considered only CLAHE fundus images for the identification of DR, and the second study considered only CECED fundus images for the identification of DR. The weighted fusion of the proposed framework presented in Figure 7 clearly revealed that the complementary fusion of CLAHE and CECED image features is capable of handling low-quality images in DR identification, achieving better recognition accuracy on both the Messidor and Kaggle datasets. Figure 8a shows the accuracy training and validation curves of the proposed methodology on both datasets, showing that the model converges smoothly with high accuracy, while Figure 8b represents the training and validation loss curves on both datasets, showing that our model achieves steady loss reduction.

**Figure 9:**
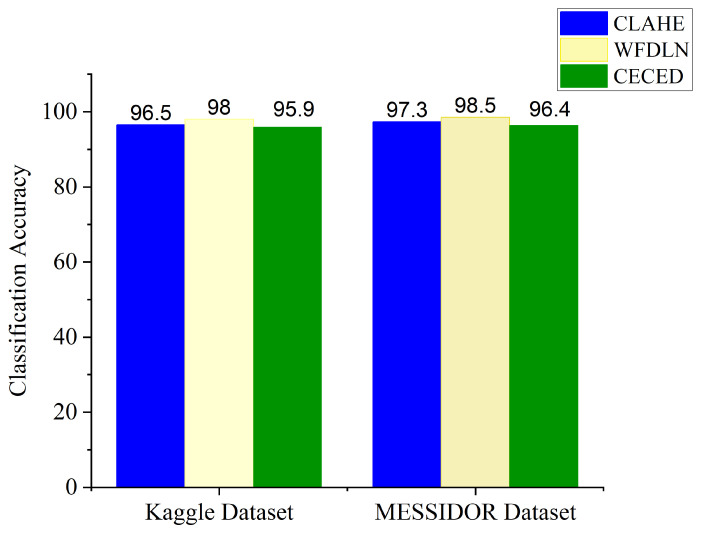
Classification accuracy for the single channels and the proposed WFDLN model.

Figure 9 presents the classification accuracy for the single channels and the proposed weighted fusion deep learning network (WFDLN). The blue bar represents the CLAHE-based channel with an Inception V3 network for the identification of DR, the yellow bar represents the proposed WFDLN for the identification of DR, and the green bar represents the CECED-based channel with the VGG-16 network for the identification of DR. From all indications, the proposed model outweighs the single-based channels, achieving 98.0% accuracy on the Kaggle dataset and 98.5% accuracy on the Messidor dataset.

**Figure 10:**
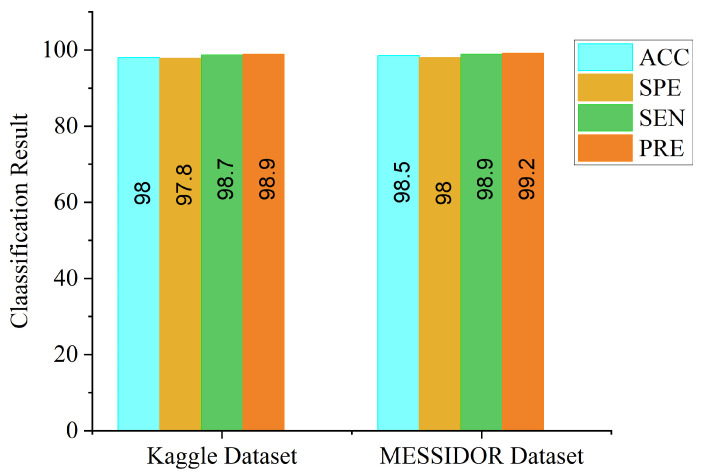
Classification result of our proposed model on Kaggle and Messidor datasets.

From Figure 9, the accuracy level of the CLAHE-based channel is higher than that of the CECED-based channel, which suggests that the contribution of CLAHE fundus images in DR identification is greater than that of CECED fundus images. We further evaluated the proposed model in terms of ACC, SPE, SEN, and PRE on both datasets, as depicted in Figure 10. It is an observable fact that the proposed model performs better on the Messidor dataset, achieving 98.5% accuracy, 98.0% specificity, 98.9% sensitivity, and 99.2% precision.

**Figure 11:**
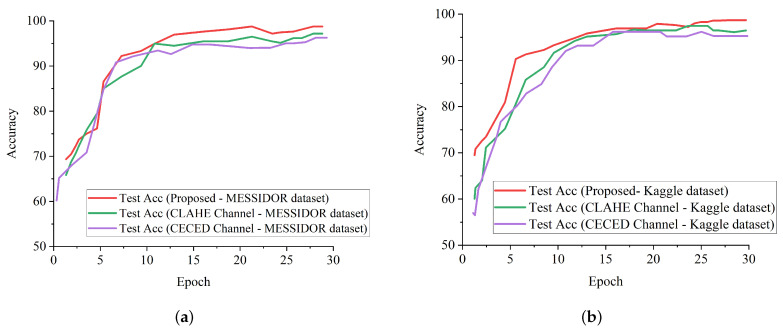
Accuracy performance report of our proposed model and single-channel models on both the Messidor and Kaggle datasets. (**a**) Test accuracy curve for our proposed model in comparison with the single channels on the Messidor dataset. (**b**) Test accuracy curve for our proposed model in comparison with the single channels on the Kaggle dataset.

Figure 11a shows the test accuracy curves of the proposed model in comparison with the single-based channels on the Messidor dataset, while Figure 11b shows the test accuracy curves of the proposed model in comparison with the single-based channels on the Kaggle dataset. It is evident that the proposed model outweighs the single-based channels on both the Kaggle and Messidor datasets.

## 5. Discussion

The performance of the proposed strategy in identifying DR in fundus images on different datasets has been presented, and the identification result for each dataset is presented in Table 3 in comparison with the single-based channels. As illustrated by the above-mentioned results, the proposed model can efficiently identify DR from non-DR fundus images. It is imperative to mention that our proposed strategy shows better generalization ability with the weighted fusion of CLAHE and CECED fundus images with a slight increase in computational time of 28.8 min.

We denoted the channel as CLAHE-based for the approach that uses Inception V3 for CLAHE fundus images, and CECED-based for the approach that uses VGG-16 for CECED fundus images. The identification results for each dataset are presented in Table 3. Our proposed model outperforms the single-based channels on all metrics, achieving 98.0% accuracy, 98.7% sensitivity, and 97.8% specificity on the Kaggle dataset, while on the Messidor dataset, the proposed model achieved 98.5% accuracy, 98.9% sensitivity, and 98.0% specificity.

We compared our proposed method with some up-to-date methods using the Messidor and Kaggle datasets. Table 4 shows that the proposed model achieved a highest AUC score of 99.1%, followed by Gulshan et al. [16] and Costa and Campilho [22] with 99.0% on the Messidor dataset. On the Kaggle dataset, the proposed model achieved a highest accuracy score of 98.0%, as seen in Table 5. Mansour et al. [25] reported a higher sensitivity score of 100% than the proposed model; however, the proposed model achieved the same AUC value of 99.0% with Mansour et al. [25].

In terms of accuracy, the proposed model achieved a highest score of 98.0%, indicating the superiority of our proposed model for DR identification. The competitive advantage of our proposed method is attributed to the complementary fusion of different channels of fundus images. It is worth mentioning that different deep learning models will perform differently under different conditions. In order to select the best-performing pre-trained model for our proposed weighted fusion deep learning framework, we conducted an ablation study using different transfer learning models, pre-trained on the ImageNet dataset.

Table 6 presents the results obtained from the experiments using our proposed framework with various pre-trained networks on the Messidor dataset. From the experimental result, the VGG-16 network shows better performance in extracting features from CECED fundus images compared to CLAHE fundus images, achieving 96.4% accuracy, 97.1% sensitivity, and 96.0% specificity. Inception V3 showed a significant improvement in performance in extracting features from CLAHE fundus images compared to CECED fundus images, achieving 97.3% accuracy, 97.8% sensitivity, and 97.0% specificity.

Table 7 shows the results of the experiments on the Kaggle dataset using our proposed framework with several pre-trained networks. According to the results of the experiments, the VGG-16 network performed better in extracting features from CECED fundus images than CLAHE fundus images, with 95.9% accuracy, 96.5% sensitivity, and 95.3% specificity. Inception V3 performed significantly better in extracting features from CLAHE fundus images than CECED fundus images, attaining 96.5% accuracy, 96.8% sensitivity, and 95.5% specificity.

**Figure 12:**
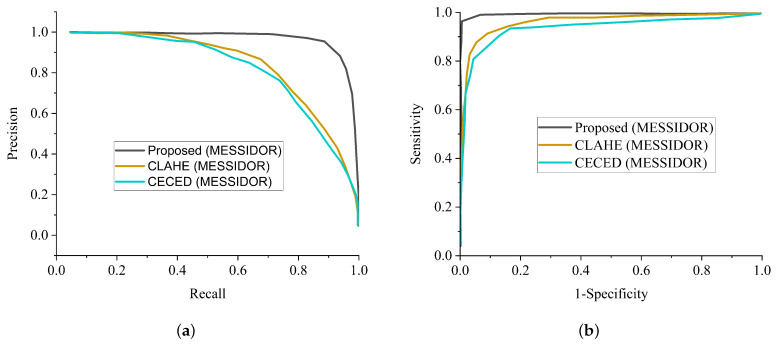
Comparison report of our model with single-channel models on the Messidor dataset. (**a**) Precision-recall curve for our proposed model in comparison with a single-channel model on the Messidor dataset. (**b**) ROC curve for our proposed model in comparison with a single-channel model on the Messidor dataset.

**Figure 13:**
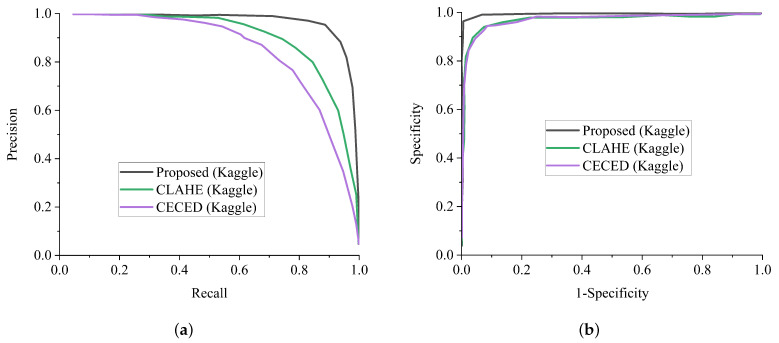
Comparison report of our model with single-channel models on the Kaggle dataset. (**a**) Precision-recall curve for our proposed model in comparison with the single-channel model on the Kaggle dataset. (**b**) ROC curve for our proposed model in comparison with the single-channel model on the Kaggle dataset.

In general, AlexNet showed the worst performance in all the metrics, followed by MobileNet in extracting features from CECED and CLAHE fundus images on both the Messidor and Kaggle datasets. For diagnosing sensitive conditions like diabetic retinopathy, it is important that we adopt a precision-recall curve to measure the mean average precision of the proposed model and the ROC curve as a method to measure the overall accuracy. Figure 12a shows the precision-recall curve and Figure 12b shows the ROC curve for the single channels and the proposed WFDLN model on the Messidor dataset. Similarly, the precision-recall curve and the ROC curve for the single channels and the proposed WFDLN model on the Kaggle dataset are presented in Figure 13a,b, respectively.

**Figure 14:**
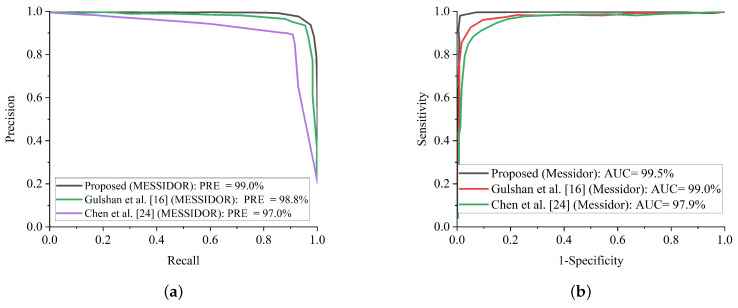
Comparison report of our model with single-channel models on the Kaggle dataset. (**a**) Precision-recall curve for our proposed model in comparison with some selected state-of-the-art methods on the Messidor dataset. (**b**) ROC curve for our proposed model in comparison with some selected state-of-the-art models on the Messidor dataset.

**Figure 15:**
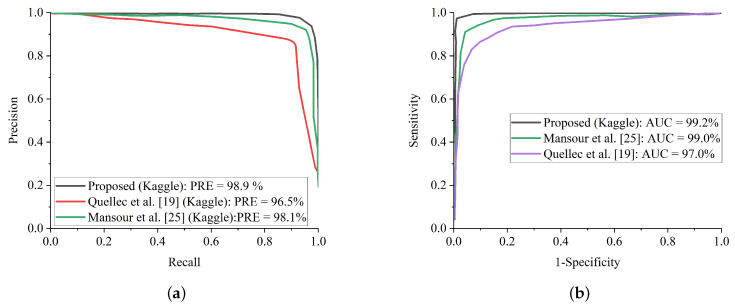
Comparison report of our model with some selected state-of-the-art methods on the Kaggle dataset. (**a**) Precision-recall curve for our proposed model in comparison with some selected state-of-the-art methods on the Kaggle dataset. (**b**) ROC curve for our proposed model in comparison with some selected state-of-the-art methods on the Kaggle dataset.

Additionally, some of the fundus images were blurred with missing details, which could have impeded the proposed model from extracting and training meaningful features. Fortunately, the benefit of improving the low quality of fundus images using CLAHE and CECED pre-processing techniques characterizes high representation details of the fundus images with observable trainable features. The proposed WFDLN achieved a satisfactory performance in identifying DR, as we went a step further to compare our proposed model with some selected up-to-date frameworks in terms of precision-recall and ROC. Figure 14a presents the precision-recall curve for the selected up-to-date models and the proposed WFDLN model on the Messidor dataset, while the ROC curve is presented in Figure 14b. Similarly, the precision-recall curve on for the selected up-to-date models and our WFDLN model on the Kaggle dataset was presented in Figure 15a, while the ROC curve was depicted in Figure 15b. It is worth mentioning that all the models were trained on the same dataset for fair comparison.

From all indications, the proposed WFDLN surpasses other frameworks in the aspect of precision-recall and ROC curves, especially in handling low-quality fundus images. The precision-recall graphs reveal that our proposed model’s curves are closest to the graph’s upper right corner with the largest area, implying that it has higher precision and sensitivity. Similarly, the ROC graphs show that our proposed model’s curve was closest to the top-left corner of the graph and had the highest AUC, indicating that it has higher sensitivity and specificity. More importantly, the stated result in terms of ROC and precision-recall can assist expert ophthalmologists in striking a balance between accuracy and precision, as described above.

Even though this study achieved a high level of accuracy in diagnosing DR, it does have certain drawbacks. This suggested strategy, which has high classification accuracy in both Messidor and Kaggle DR datasets, might not obtain exactly the same classification accuracy in another medical dataset. The reason is because the images of various datasets differ owing to differences in labeling, noise, and other factors. To solve this challenge, the AI should be taught to utilize images acquired at various times and locations. Aside from the diversity of data, the allocation of the data classes is also significant. A disparity in class sizes has a detrimental impact on training. The accuracy of categorization was also affected by the different data augmentation strategies employed to correct the imbalance. Another disadvantage is that using the weighted fusion technique requires more processing time as compared to single-channel models while improving the classification accuracy. In light of these constraints, tests will be conducted in the future employing a wider range of images and possibly employing various optimization strategies that are more efficient in terms of computational time.

## 6. Conclusions

Our article proposed a DR identification technique based on weighted fusion capable of processing CLAHE and CECED fundus scans concurrently. We mentioned that these channels are fused to capture meaningful details from fundus images and achieve higher levels of identification accuracy. The strategy of weighted fusion was utilized to take complete advantage of the visual features that have been captured from the different channels. The proposed WFDLN model handles the problem of low-quality fundus images by fusing the weighted features generated from CLAHE and CECED pre-processing stages. The VGG-16 model was fine-tuned to extract features of diabetic-related retinopathy from CECED fundus images, while the Inception V3 model was fine-tuned to extract features of diabetic-related retinopathy from CLAHE fundus images. Furthermore, both features were merged by utilizing the weighted fusion strategy in order to take advantage of the complementary retinopathy information. Softmax was introduced as the classifier to obtain the fused features. By fusing channels of complementary attributes in a weighted manner, our proposed model outweighs several up-to-date models. The evaluation results show that the proposed model achieves better performance with an accuracy of 98.5%, sensitivity of 98.9%, and specificity of 98.0% for the Messidor dataset, whereas on the Kaggle dataset, the proposed model reports an accuracy level of 98.0%, sensitivity of 98.7%, and specificity of 97.8% than just using the single channels. From the comparative results of the other established methods, it is confirmed that the proposed WFDLN model achieved a state-of-the-art identification accuracy level of 98.5% on the Messidor dataset and 98.0% on the Kaggle dataset, which makes it a robust and efficient identification solution for low-quality fundus images. These findings could efficiently help ophthalmologists determine whether RDR is present or not while saving time.

## Figures and Tables

**Table 1:** Description of Messidor dataset.

DR Stages	Details	Number	Label
Healthy	Zero abnormalities	548	Normal
Mild NPDR	Microaneurysms	152	Stage 1
Moderate NPDR	Few microaneurysms	246	Stage 2
Severe NPDR	Venorous beading + Intraretinal microvascular abnormality		
PDR	Vitreous/Pre-retinal hemorrhage	254	Stage 3

**Table 2:** Description of Kaggle dataset.

Class	Number	Label
No DR	25,810	0
Mild DR	2443	1
Moderate DR	5292	2
Severe DR	873	3
Proliferative DR	708	4

**Table 3:** Comparison of our proposed model with single channels.

Model	Kaggle Dataset						Messidor Dataset					
	ACC (%)	SEN (%)	SPE (%)	PRE (%)	AUC (%)	Time (Min)	ACC (%)	SEN (%)	SPE (%)	PRE (%)	AUC (%)	Time (Min)
CLAHE-based channel	96.5	97.1	96.0	97.3	97.5	18.5	97.3	97.8	97.0	98.0	98.7	18.0
CECED-based channel	95.9	96.5	95.3	96.4	97.0	24.0	96.4	97.1	96.0	96.9	97.1	24.3
**WFDLN**	**98.0**	**98.7**	**97.8**	98.9	99.2	28.8	**98.5**	**98.9**	**98.0**	99.0	99.5	28.8

**Table 4:** Result comparison of our proposed model with up-to-date methods on the Messidor dataset for fundus classification. EEL stands for end-to-end learning.

Model	Training Type	Method (%)	Process Type (%)	ACC (%)	AUC (%)	SEN (%)
Gulshan et al. [16]	CNN	Transfer Learning	Fundus Classification	-	99.0	87.0
Costa and Campilho [22]	SURF + CNN	EEL	Fundus Classification	-	99.0	-
Gargeya and Leng [17]	CNN	EEL	Fundus Classification	-	94.0	-
Wang et al. [23]	Zoom	EEL	Fundus Classification	91.1	95.7	-
Chen et al. [24]	SI2DRNet	EEL	Fundus Classification	91.2	96.5	87.0
WFDLN	CNN	Transfer Learning	Fundus Classification	98.5	99.1	98.9

**Table 5:** Result comparison of our proposed model with up-to-date methods on Kaggle dataset for fundus classification. EEL stands for end-to-end learning.

Model	Training Type	Method (%)	Process Type (%)	ACC (%)	AUC (%)	SEN (%)
Mansour et al. [25]	AlexNet + SVM	Transfer Learning	Fundus Classification	97.9	99.0	100
Quellec et al. [19]	CNN	EEL	Fundus Classification	-	95.5	-
Colas et al. [26]	CNN	EEL	Fundus Classification	-	94.6	96.2
Pratt et al. [27]	CNN	EEL	Fundus Classification	75.0	-	95.0
Jinfeng et al. [28]	CNN	Transfer Learning	Fundus Classification	80.3	-	-
WFDLN	CNN	Transfer Learning	Fundus Classification	98.0	99.0	98.7

**Table 6:** Results obtained on the Messidor dataset using different pre-trained models on our proposed WFDLN.

Model	CLAHE-Based Channel			CECED-Based Channel		
	ACC	SEN	SPE	ACC	SEN	SPE
AlexNet	89.5	91.6	88.0	88.9	88.0	87.2
VGG-16	95.2	96.3	94.9	96.4	97.1	96.0
ResNet-50	95.0	95.5	94.5	95.7	95.9	95.2
ResNet-101	93.9	94.5	94.1	94.5	95.0	93.2
MobileNet	92.2	92.8	91.2	92.8	93.1	92.4
DenseNet	94.5	93.7	92.2	94.2	94.6	94.9
Inception V3	97.3	97.8	97.0	95.8	96.0	95.3
Xception	91.9	92.6	89.6	91.3	92.8	90.7

**Table 7:** Results obtained on the Kaggle dataset using different pre-trained models on our proposed WFDLN.

Model	CLAHE-Based Channel			CECED-Based Channel		
	ACC	SEN	SPE	ACC	SEN	SPE
AlexNet	89.3	90.5	88.1	88.2	87.8	88.0
VGG-16	92.9	93.4	91.7	95.9	96.5	95.3
ResNet-50	93.2	96.0	95.4	92.7	93.3	92.1
ResNet-101	95.0	96.5	95.2	94.2	95.4	94.6
MobileNet	95.8	96.2	94.2	93.8	92.2	91.9
DenseNet	91.7	92.9	90.3	91.4	92.1	92.5
Inception V3	96.5	96.8	95.5	95.1	96.0	94.7
Xception	94.4	95.1	94.0	93.6	94.9	92.8

## Data Availability

The datasets As cited within the text As reference [33,34] used in this study are available at: https://www.adcis.net/en/third-party/Messidor/ and https://www.kaggle.com/c/diabetic-retinopathy-detection/data (accessed on 1 January 2022).
